# Performance, Cost-Effectiveness, and Representativeness of Facebook Recruitment to Suicide Prevention Research: Online Survey Study

**DOI:** 10.2196/18762

**Published:** 2020-10-22

**Authors:** Sylvia Lee, Michelle Torok, Fiona Shand, Nicola Chen, Lauren McGillivray, Alexander Burnett, Mark Erik Larsen, Katherine Mok

**Affiliations:** 1 Black Dog Institute University of New South Wales Sydney Australia

**Keywords:** research subject recruitment, social media, suicide

## Abstract

**Background:**

Researchers are increasingly using social media advertisements to recruit participants because of their many advantages over traditional methods. Although there is growing evidence for the effectiveness and cost-effectiveness of social media recruitment in the health sciences, no studies have yet examined this in the context of suicide prevention, which remains to be a highly stigmatized and sensitive topic.

**Objective:**

This study aims to recruit a general community sample to complete a survey on suicide literacy, stigma, and risk via Facebook advertisements. Specifically, we aim to establish the performance of the advertisements, cost-effectiveness, sample representativeness, and the impact of gender-specific advertising on recruiting men into the study.

**Methods:**

From June 2017 to March 2019, we released Facebook advertisements targeted at adults 18 years or older, residing in the New South Wales (NSW) trial or control regions, and involved in the LifeSpan suicide prevention trial. Cost-effectiveness was examined descriptively using metrics provided by Facebook. Chi-square analyses were conducted to determine demographic differences between our sample and the general NSW population as well as the impact of gender-specific advertisements on gender engagement.

**Results:**

The 14 Facebook advertisement campaigns reached a total of 675,199 people, yielding 25,993 link clicks and resulting in 9603 individuals initiating the survey (7487 completions) at an overall cost of Aus $2.81 (US $2.01) per participant. There was an overrepresentation of younger (*P*=.003), female (*P*=.003), highly educated (*P*<.001) participants and mental health conditions (*P*<.001) compared with the total NSW population. The use of male-specific advertisements resulted in a significantly higher proportion of men completing the survey relative to gender-neutral advertisements (38.2% vs 24.6%; *P*<.001).

**Conclusions:**

This study demonstrates the potential of Facebook to be an effective, low-cost strategy for recruiting a large sample of general community participants for suicide prevention research. Strategies to improve sample representativeness warrant further investigation in future research.

## Introduction

Suicide is a significant and complex public health issue, with more than 800,000 deaths per annum [1]. It remains to be a largely unspoken and stigmatized topic in many countries, and this stigma may, in part, prevent individuals not only from seeking help [2-4] but also from participating in research which may advance our understanding of risk and protective factors.

To develop suicide prevention initiatives that address the needs of individuals and communities, measuring risk, suicide literacy, and stigma is necessary [5,6]. However, recruiting community-based participants into health survey research, specifically mental health, has historically been challenging [6-9]. Challenges include logistical barriers, such as geography, transportation, and time constraints [7], but also more complex personal factors, such as mistrust in research programs, concerns about privacy and confidentiality [10], and stigma surrounding mental health issues [11].

The growing reach of web-based social media platforms offers a new opportunity to enhance recruitment for suicide prevention research purposes [12]. For example, as of November 2019, Facebook had 2.45 billion active users per month [13], of which 15 million are Australians [14], positioning it as the largest social media platform globally. A systematic review conducted by Thornton et al [15] reports that more than 100 health-focused research studies have used Facebook as a recruitment strategy in the past decade, demonstrating its emerging appeal. Previous studies have used Facebook to not only recruit participants from the general community [9] but also to target audiences based on specific demographics, regional characteristics, or user interests [12,16]. Owing to Facebook’s added advantages of anonymity and confidentiality, it has demonstrated potential in recruiting hard-to-reach individuals for research surrounding highly sensitive and stigmatizing issues, such as human immunodeficiency virus [17], mental health [9,12], sexuality [18], and substance use [19].

Another advantage of social media recruitment is its potential cost-effectiveness. A review of health research studies recruiting via Facebook advertisements found this approach to be considerably more affordable (an average of US $12.53, SD $23.16 per participant) [15] than traditional recruitment strategies, such as flyers, newspaper advertising, or face-to-face recruitment [9] and other web-based (non–social media) advertising strategies (up to US $66.15 per participant) [15,19,20]. However, the literature on cost-effectiveness has largely focused on substance use and smoking cessation [12], and no research studies have yet examined whether Facebook is a cost-effective recruitment approach in the field of suicide prevention. There may be variability in the cost-effectiveness of Facebook recruitment across research areas, particularly those perceived as more stigmatized, warranting the replication of such findings in the context of suicide prevention research.

Although Facebook has many potential advantages over traditional methods of participant recruitment, there is limited research examining the representativeness of participants recruited via Facebook [15,16]. Characteristics or populations that were most often reported to be overrepresented included younger participants [9,21,22], women [15,23], and those who were well educated [8,21,24]. The issue of representativeness is particularly pertinent in the conduct of a suicide prevention study, as certain populations (eg, men, older adults, and those from cultural and linguistically diverse minority groups) have a higher risk of suicide [25,26] but are less likely to participate in research [12,23]. The lower levels of research participation among these groups may be due to higher levels of stigma of suicide [27], perceived nonrelevance, or poorer internet access and social media usage [28]. Given that the use of Facebook recruitment in mental health research is increasing, it is important to investigate sample representativeness and whether specific advertising strategies can enhance representativeness, such as the use of gender-specific wording or imagery. To the best of our knowledge, this is the first study to report on whether the use of male-specific advertisements increases the response rates of men in mental health research.

### Aims

To date, no studies have examined whether targeted, paid Facebook advertising can be used to recruit a large sample of community participants into suicide prevention research. Accordingly, this study aims to advance our understanding of the usefulness of Facebook advertising as a means of recruiting a general community sample into a survey on suicide risk, literacy, and stigma. The survey was delivered as part of a multilevel suicide prevention trial known as LifeSpan in New South Wales (NSW) [29]. LifeSpan is being implemented at 4 sites (Newcastle, Illawarra Shoalhaven, Central Coast, and Murrumbidgee), with 3 corresponding control sites: South Western Sydney, Nepean Blue Mountains, and Western NSW. The study addresses gaps in our understanding of how to improve sample representativeness by testing gender-specific advertisements against gender-neutral advertisements to examine whether this approach results in an increased rate of survey participation by men. The specific aims of this study were to (1) determine the cost-effectiveness of Facebook as a recruitment tool for suicide prevention research in the general population; (2) determine the performance of Facebook advertisements with respect to reach, views, and survey initiations and completions; (3) examine whether a representative community sample can be recruited through Facebook; and (4) examine whether gender-specific advertisements increase the rate of male participation.

### Methods 

#### Study Design

The survey was delivered using a longitudinal panel design in which a group of community members at the LifeSpan intervention and control sites were followed up for over 2 years at multiple time points based on the formal implementation period of the trial (T0: baseline recruitment at 3 months before LifeSpan being delivered, T1: at 12 months postbaseline, and T2: at 24 months postbaseline). To account for potential attrition in the first panel, a second panel was recruited at the T1 time point of the first panel. This study examines the cost-effectiveness and feasibility of Facebook recruitment based on data from the baseline recruitment time point (T0) of the first and second panels.

#### Participants

**Figure 1:**
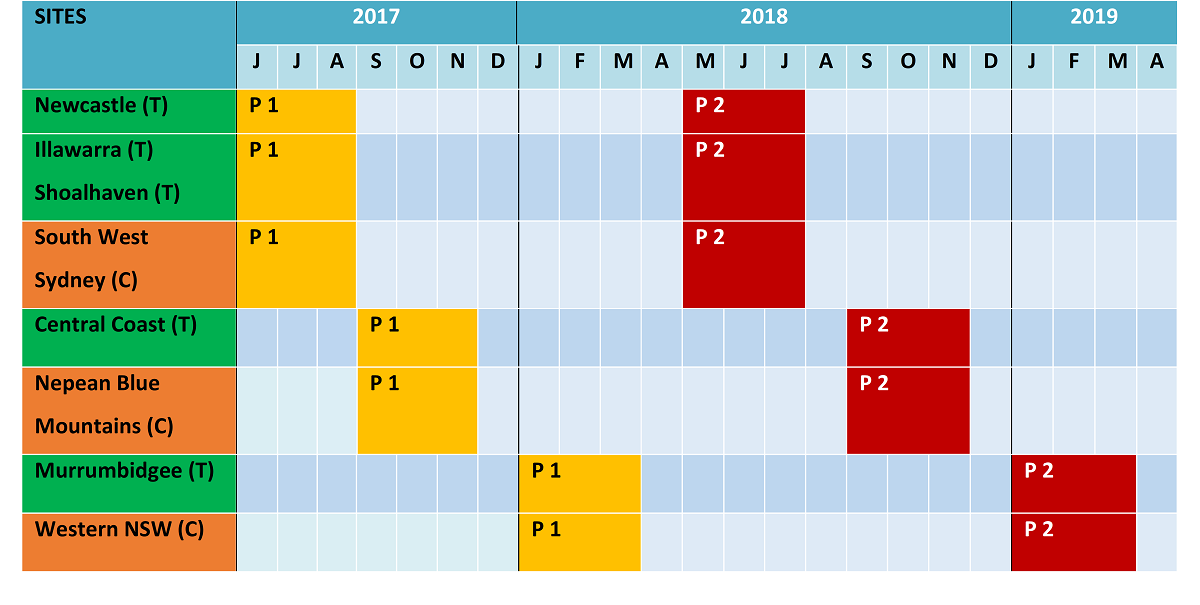
Baseline recruitment for Panel 1 and Panel 2 trial (T) and control (C) sites.

In total, 14 unique Facebook advertisement campaigns were used to recruit participants from June 2017 to March 2019, over a 68-week recruitment period. The duration of each campaign ranged from 4 weeks to 16 weeks (Figure 1), and the duration was determined by an algorithm in Facebook based on the advertisements that were being viewed.

The Facebook advertisements were targeted at individuals whose profiles matched the following inclusion criteria: (1) 18 years or older, (2) residing in LifeSpan NSW trial regions or corresponding control regions, and (3) able to competently read and understand English.

There were no specific exclusion criteria.

#### Procedure

For the first panel (June 2017 to March 2018), targeted, paid Facebook advertisements containing a short headline (eg, “Lend a Voice to Suicide Prevention” or “Share a Voice to Help Out”) were displayed to all users aged 18 years and older residing in one of the targeted locations. These advertisements included a generalized image and a brief description of the study.

For the second panel (May 2018 to March 2019), an additional set of Facebook advertisements was included with male-focused imagery (eg, photo of a man) and male pronouns in the advertisement wording (“voice of local blokes to help make it better”). The advertisements were also targeted specifically at men in each recruitment location according to Facebook user profiles. In panel 2, a Facebook pixel was also configured in the entry page of the survey link to improve conversion rates by tracking target user activity and remarketing the advertisements to those who were interested (clicked on survey link but did not complete the survey).

Once individuals clicked on the Facebook advertisements, they were directed to a web-based study portal where interested persons completed a brief eligibility screener. Eligible participants were directed to read a participant information sheet, and by starting the web-based survey, individuals consented to being involved in the study. Eligibility was determined by participants’ age, postcode, and suburb, which had to map to the trial or control sites. Participants who were eligible to participate were provided with a link to a web-based survey. The survey included questions about basic demographic parameters (eg, age, gender, highest level of education, employment status, language spoken at home, marital status, ethnicity) and a series of suicide and help-seeking scales (Suicidal Ideation Attributes Scale, Distress Questionnaire-5, Stigma of Suicide Scale, Literacy of Suicide Scale, and Actual Help-Seeking Questionnaire) to measure the primary outcomes of interest for the broader LifeSpan study. At the end of the survey, participants were asked to provide their email ID if they consented to being contacted for follow-up research. No incentives were offered to the participants. To prevent duplicate entries, only one survey per internet protocol address per panel was accepted.

#### Participant Safety

Given the potentially distressing nature of the study, in the consent process, participants were provided with a clear outline of the aims of the research and the types of questions they would be asked as well as a list of helplines and help resources. These help resources were listed again at the end of the survey. Participants were reminded periodically throughout the survey that if they felt distressed at any time, they could immediately discontinue and contact their GP, one of the help resources provided, or a member of the research team for further assistance. This safety procedure was approved by the Hunter New England Local Health District Human Research Ethics Committee (approval number 16/09/21/4.05) and is consistent with the 2007 National Statement on Ethical Conduct in Human Research [30].

### Measures

For the purposes of this study, the primary outcomes of interest were cost-effectiveness of Facebook recruitment, sample representativeness, and gender-specific engagement.

The effectiveness of the Facebook advertisements across the first and second panels at baseline (T0) recruitment was assessed using the following performance metrics provided by Facebook:

- *Reach:* number of Facebook users who saw the advertisement.
- *Link Clicks:* number of clicks the advertisement received.
- *Click through rate (CTR):* percentage of link clicks per reach.
- *Surveys started:* the total number of people who initiated the survey (partial survey completions+completions).
- *Completions:* number of completed surveys.
- *Conversion rate:* percentage of surveys started per link click.
- *Completion rate:* percentage of surveys completed per link click.
- *Cost per completion:* total costs of advertisements divided by number of survey completions (cost-effectiveness metric).

Cost-effectiveness was defined as the cost per completed survey and reported in Australian dollars. This was calculated by dividing the total cost spent on advertising across the entire recruitment period by the number of survey completions.

The representativeness of our sample was evaluated based on the self-reported sociodemographic characteristics, such as gender, age, language spoken at home, and highest level of education. These characteristics were compared with those of the total NSW population using nationally representative 2016 Australian census data [31] and mental health data from the NSW results of the 2017-2018 National Health Survey [32].

The performance of the gender-specific advertisements was measured by comparing the proportion of men and women who partially completed or completed the survey in panels 1 and 2.

### Statistical Analysis

The results were analyzed using IBM SPSS statistical software version 25.0 [33]. Data were treated as missing if a question was skipped or not answered. The effectiveness and cost-effectiveness of the Facebook advertisements were analyzed descriptively using the performance metrics described earlier and by calculating the cost per completed survey, adopting a methodology similar to that of an earlier seminal study in this area [9]. Sample representativeness was assessed by comparing the demographic characteristics of participants who completed our survey with those of the general NSW population. Differences in proportions of characteristics were analyzed using the chi-square goodness-of-fit statistic to determine whether the survey initiators in our sample were consistent with the expected distribution in the NSW population. Differences between advertisement illustrations and the gender of participants were analyzed using the chi-square tests of independence, with odds ratio and 95% CIs reported. The alpha value was set at .05 for all analyses.

### Ethics Approval

The Hunter New England Human Research Ethics Committee (HREC/16/HNE/399) approved this study. The privacy of all participants was maintained throughout the study. Participant data were not accessible to Facebook, and participants’ Facebook data were not accessible to the researchers. The data provided by Facebook to the research team were at an unidentifiable aggregate level. For example, researchers were able to see the (ie, demographics [age, gender]) characteristics of the people who clicked on the advertisements. Any identifiable information collected was only used for the purposes of contact for follow-up. No individuals were identified in the reporting of results.

## Results

### Facebook Advertising Campaign—Recruitment Rate

During the recruitment period (June 2017 to February 2019), the Facebook advertisements reached a total of 675,199 people, yielding 25,993 link clicks (CTR=3.85%). Of those who clicked on the link, 7478 (77.88%) people completed the web-based survey (n=3224 in panel 1; n=4254 in panel 2) and 2125 (22.12%) people partially completed the survey (n=786 in panel 1; n=1339 in panel 2. Table 1 describes the performance of the advertisements according to Facebook metrics across the first and second panels of recruitment.

**Table 1:** Facebook recruitment advertisement performance across all sites.

Facebook metrics	Panel 1	Panel 2	Overall
Reach, n (%)	413,742 (61.28)	261,457 (38.72)	675,199 (100)
Link clicks, n (%)	15,291 (58.83)	10,702 (41.17)	25,993 (100)
Click through rate, %	3.70	4.09	3.85
Surveys started^a^, n (%)	4010 (41.76)	5593 (58.24)	9603 (100)
Surveys completed, n	3224 (43.11)	4254 (56.89)	7478 (100)
Conversion rate, %	26.22	55.26	36.94
Completion rate, %	21.08	39.75	28.77

^a^Survey started=partially completed surveys+completed surveys; click through rate=percentage of link clicks divided by reach; conversion rate=percentage of completions divided by link clicks; completion rate=percentage of completions divided by link clicks.

### Cost-Effectiveness

Table 2 displays the cost-effectiveness of Facebook advertisements in this study and provides comparisons with earlier studies. The overall expense was Aus $20,997.97 (US $15,055.97), with an average cost per participant of Aus $2.81 (US $2.01), with specific costs per panel described in Table 2. The costs reported for this study were commensurate with those of other studies that have recruited Australian general community adults (R: Aus $0.55-Aus $9.82 [US $0.39-$7.04]) but lower than those of studies that were conducted primarily overseas [12,27,28]. The Australian-based studies all recruited samples from the general population, whereas two of the international studies [12,27] recruited specific populations where subthreshold or full diagnostic mental health symptoms were the eligibility criteria.

**Table 2:** Cost-effectiveness comparison of this study with prior mental health studies using social media recruitment methods.

Studies		Recruitment target	Country from which the participants were recruited	Total cost of advertisements Aus $ (US $)	Completed surveys (n)	Cost per participant Aus $ (US $)
**This study**						
	First panel	Suicide in adults aged ≥18 years from general community	Australia/NSW	14,497.97 (US $10,388.17)	3224	4.50 (US $3.23)
	Second panel	Suicide in adults aged ≥18 years from general community	Australia/NSW	6500.00 (US $4,660.63)	4254	1.53 (US $1.10)
**Prior studies**						
	Batterham (2014)—round 1 recruitment [9]	Mental health; adults aged ≥18 years from general community	Australia	12,600 (US $9034.45)	1283	9.82 (US $7.04)
	Batterham (2014)—round 2 recruitment [9]	Mental health; adults aged ≥18 years from general community	Australia	920 (US $659.66)	610	1.51 (US $1.08)
	Choi et al (2017) [34]	Mental health in men aged ≥18 years from general community	Australia	500 (US $358.50)	398	0.55-3.85 (US $0.39-$2.76)
	Chu and Snider (2013) [35]	Posttraumatic stress disorder in Canadian youth (aged 15-24 years) affected by violence	Canada	1508.26 (US $1081.45)	88	17.13 (US $12.28)
	Morgan et al (2013) [12]	Adults with subthreshold symptoms of depression	Australia, New Zealand, the United Kingdom, Ireland, Canada, the United States	696.15 (US $499.15)	35	19.89 (US $14.26)
	Youn et al (2013) [36]	Depression in students across 5 colleges	The United States	338.8 (US $242.93)	20	16.94 (US $12.15)

### Sample Representativeness

Table 3 compares the characteristics of our sample with those of the total NSW population from which the survey respondents were recruited. Those who completed the survey (*completers*) and the survey initiators differed significantly on all variables compared with the referent NSW population. Compared with the NSW population, our sample had a significantly higher proportion of women (66.5%) and an underrepresentation of older adults (≥60 years) in our sample (14.2%). Our samples were also significantly more likely to have tertiary qualifications and to primarily speak English at home than the broader NSW population. Significantly elevated rates of mental health conditions were reported by our sample.

**Table 3:** Demographic and mental health characteristics of survey respondents compared with those of the New South Wales general population.

Characteristics		Partial completers, n %	Completers, n %	Survey initiators^a^, n %	NSW^b^ general population, %	Completers versus NSW		Survey initiators versus NSW		
						Chi-square (*df*; number of participants included in the sample)	*P* value		Chi-square (*df*)	*P* value
**Gender**						10.24 (1, n=8821)	.002		9.01	.003
	Female	894 (62.5)	4971 (67.31)	5,865 (66.49)	3,794,217 (50.72)					
	Male	537 (37.5)	2419 (32.69)	2,956 (33.51)	3,686,014 (49.28)					
**Age (years)**						11.75 (1, n=8839)	.003		11.75	.003
	18-34	448 (31.1)	2,139 (28.91)	2,587 (29.27)	1,647,194 (27.5)					
	35-59	757 (52.6)	4,241 (57.32)	4,998 (56.54)	2,450,605 (43.5)					
	≥60	235 (16.3)	1,019 (13.77)	1,254 (14.18)	1,637,690 (29.0)					
**Language at home**						9.47 (1, n=9524)	.002		7.67	.006
	English only	2,122 (100)	7,274 (98.27)	9,396 (98.66)	5,126,633 (87.45)					
	Language other than English	0	128 (1.7)	128 (1.3)	735, 563 (12.55)					
**Highest education^c^**						22.76 (1, n=8885)	<.001		19.38	<.001
	Less than year 12	220 (15.2)	759 (10.2)	979 (11.0)	1,479,305 (32.54)					
	Year 12	240 (16.6)	962 (12.9)	1,202 (13.52)	930,654 (20.47)					
	Tertiary qualification	986 (68.2)	5,718 (76.86)	6,704 (75.45)	2,135,805 (46.98)					
**Mental health condition**						149.71 (1, n=8884)	<.001		145.54	<.001
	Yes	976 (67.5)	4,969 (66.79)	5,945 (66.9)	1,428, 724 (19.09)					
	No	469 (32.5)	2,470 (33.21)	2,939 (33.08)	6,051,507 (80.91)					

^a^Survey initiators=partial completers+completers.

^b^NSW: New South Wales.

^c^Year 12 is the final year of high school equivalent according to the Australian education system; note: for all categories missing or unknown responses were excluded from percentage calculations.

### Gender-Specific Advertisement Effects

Multimedia Appendix 1 shows an example of the gender-neutral advertisements used in panel 1 recruitment (advertisement 1 and advertisement 2) as well as examples of advertisements targeted toward men using different language and imagery (advertisement 3 and advertisement 4) in panel 2.

Table 4 shows the proportion of male and female participants recruited for each of the 2 panels (panel 1: gender-neutral advertisements; panel 2: gender-focused advertisements). The chi-square analysis showed significant gender differences in recruitment responses across the 2 panels for survey completions and for partial completions, with an increase observed in the proportion of male respondents in panel 2 and a decrease in female respondents relative to panel 1.

**Table 4:** Gender of participants across panels of recruitment.

Survey completion status		First panel recruitment: gender-neutral advertisements	Second panel recruitment: gender-specific advertisements	Chi-square (*df*; number of participants included in the sample)	*P* value	OR (95% CI)
**Survey completers, n (%)**				159.4 (1, n=7390)	<.001	1.92 (1.25-1.35)
	Male	793 (24.6)	1626 (38.22)			
	Female	2402 (74.50)	2569 (60.39)			
	Other/unknown^a^	29 (0.9)	59 (1.4)			
	Total	3224 (100)	4254 (100)			
**Partial completions, n (%)**				17.21 (1, n=1431)	<.001	1.63 (1.30-2.05)
	Male	157 (20.0)	380 (28.4)			
	Female	360 (45.8)	534 (39.9)			
	Other/unknown^a^	269 (34.2)	425 (31.7)			
	Total	786 (100)	1 339 (100)			

^a^This group is not included in chi-square analyses; partial completions=started surveys but did not finish.

## Discussion

### Performance and Cost-Effectiveness Findings

This is the first study to examine the cost-effectiveness and representativeness of Facebook as a recruitment medium for suicide prevention research. Facebook was found to be an effective platform for recruiting a large sample of community members at a relatively low cost (average cost of Aus $2.81 [US$2.01] per survey completed). The overall cost per participant compared favorably with prior mental health studies that recruited similar populations, that is, adults from the general population [9,35], and costs were much lower than those of studies that recruited more specialized populations, with more stringent eligibility criteria, such as the presence of a particular health condition. Smaller pools of participants from which to draw from may prolong recruitment periods, resulting in larger advertising expenses over longer durations to meet recruitment targets. The estimated costs per survey completion were also lower than those reported for traditional recruitment methods, such as postal surveys or telephone calls, which are reported to range in cost from Aus $19.10 [9] to Aus $24.75 [37] per survey. This study adds to a growing body of evidence that supports the cost-effectiveness of social media recruitment strategies, particularly for general community samples.

The advertising campaign also had strong positive engagement, with more than 25,000 people clicking on the survey link. This resulted in a substantially higher CTR than the average Facebook advertising rate of 0.90% and the benchmark rate for health care industry standards (0.83%) [38], potentially highlighting the salience of the research topic. However, the large number of link clicks did not translate to a large number of survey initiations or completions, with just over two-third starting the surveys and approximately one-fourth completing them. This divergence between link clicks and survey completion is consistent with prior research, which has found that Facebook users tend to click advertisement links on impulse and lack the commitment to see the task through to its end [16,39]. In our second panel, however, we observed an increase in conversion rates and lower cost per participant, despite the lower total cost spent compared with the first panel. This could partly be because of an increased interest in, or awareness of, the LifeSpan trial, which was in its second year of implementation at the time of recruitment of the second panel. This improved result could also be because of the installation of a Facebook pixel during the second panel of recruitment, which tracks user activity after they see the Facebook advertisement and retargets users who are interested (ie, visited the Facebook page or clicked on the survey link but did not complete the survey). The pixel also optimizes conversions by automatically allocating more money to advertisements with greater success rates (higher conversions) and less money to advertisements that are performing poorly. As such, the use of Facebook pixels may be a promising approach to social media recruitment strategies in future studies.

### Representativeness

The findings from this study indicated that there were significant differences in demographic and mental health characteristics of our sample and those of the target general population in NSW. Our participants were more likely to be younger, female, better educated, and less culturally diverse (eg, most participants could only speak English) compared with the overall NSW population. These findings are largely consistent with findings from prior research on general population samples [9,16,21]. Although Facebook mainly constitutes younger users, there is evidence that Facebook is gaining popularity among older user groups [40,41], suggesting that there is potential to increase engagement with older adult populations in web-based surveys over time.

The higher education levels seen in our sample are not only consistent with those reported in prior samples recruited via Facebook [42] but also similar to patterns of educational attainment in participants recruited through traditional recruitment strategies [42,43]. This suggests that, generally, people with higher education levels tend to engage in health research studies, potentially because of greater levels of health literacy and increased awareness of their importance. Although the high levels of English-speaking participants in this sample could indicate that those from culturally and linguistically diverse backgrounds may experience barriers engaging in health research, it may also simply be an artifact of our eligibility criteria, which required proficiency in English.

There were also significantly higher rates of mental health conditions in our sample compared with the general NSW population. Previous mental health research has similarly observed elevated rates of mental health problems among participants relative to the general Australian population [9]. Such overrepresentation may reflect a potential self-selection bias, with individuals experiencing mental health problems more inclined to participate in mental health research because the subject matter interests or concerns them. The degree to which these differences will have significant implications for the validity of the study depends on the research topic and design. As suicide is one of the leading causes of death among young depressed people [1], having overrepresentation of young people and people with mental health disorders may be useful in understanding risks and help-seeking behaviors for suicidality. However, in community research that is intended to understand the prevalence of stigma and awareness of suicide in the general community, such overrepresentation may be a shortcoming of the study.

The final aim was to evaluate the impact of gender-specific advertisement content on recruitment rates. Consistent with recent studies, our findings from the first panel of recruitment suggest that men are more difficult to recruit than women and are underrepresented in health research studies. The introduction of targeted, gender-specific advertisements (using colloquial language such as *blokes* in text captions) appeared to appeal to Australian men, with significant improvement in the number of men responding to the survey in panel 2 compared with panel 1. The effectiveness of using male- or female-specific imagery and/or wording has been demonstrated in only a few previous studies of social and health factors [24,35] but suggests that men are more likely to participate when they are specifically called to action. As suggested by Fenner and Garland [8], Facebook recruitment has a great potential to yield a demographically representative sample by oversampling specific subgroups of the population. In research studies where men present a higher risk of a health problem, consideration should be given to allocating a budget for the design and development of male-specific advertisements. In addition, novel techniques such as machine learning, which analyzes patterns of how men use social media compared with women and how they engage in health content on the web, might provide valuable new insight into reaching men for health research purposes using Facebook advertisements.

### Limitations

As with all studies, this study is not without limitations. Our study recruited a general population sample from selected trial and control sites in NSW, Australia. As such, the findings of this study may not be generalizable to different populations and settings. Relatedly, we acknowledge that we have only captured and reported on very few demographic and mental health factors in this study as measures or *representativeness*. The decision regarding what factors to report on for representativeness was limited by the availability of matched NSW population-level data; however, we fully acknowledge that these variables are a limited measure of *representativeness*. To fully describe the representativeness of Facebook or social media recruited samples, future studies should look to capture precise measures of socioeconomic variables, employment, sexuality, and cultural and linguistic diversities, giving careful consideration to measures that are consistent with census data or national surveys for comparability. In addition, although the advertisements had high levels of reach (exposure), comparatively fewer people went on to initiate the survey. Without person characteristic data on the group exposed (vs the survey initiators), we are not able to determine whether representativeness issues are an artifact of the archetypal Facebook user (ie, are younger, better educated persons) and therefore underrepresented groups are not seeing the advertisements (eg, older people, less educated people) or whether underrepresented groups do not want to participate in research studies. If representativeness issues are to be addressed, future research should seek to determine whether underrepresentation is because of exposure, motivational, or access-related reasons.

Furthermore, when determining the feasibility of using Facebook advertisements to recruit participants, it is worth considering that we do not know if the participants who clicked on the advertisements and completed the survey were the same people who were exposed to the Facebook advertisement campaigns. This is because Facebook recruitment is liable to uncontrolled snowballing, as participants could *share* the survey link or *tag* Facebook friends that they think would be interested in the study. Future studies that examine Facebook’s advertising to recruit participants should collect information about how an individual was exposed to the study and implement pixels to further study participant conversion patterns.

Finally, the strategy we used to improve gender (specifically male) representativeness in this study (ie, male-centric wording and masculine imagery) may not be appropriate to redress imbalances in the participation of other demographic groups, such as the poorly educated or older adults. For such groups, access to social media itself might be a key barrier to participation in web-based surveys. Strategies to improve engagement with these groups might include targeting the family or peer social media networks of these groups to promote research studies and assist these groups in participating. Understanding what strategies work to improve participation in groups of interest is an area that warrants investigation in future studies.

The findings indicate that advertising suicide prevention research using Facebook is a feasible and cost-effective way to recruit a community-based sample. Preliminary evidence suggests that gender-specific advertisements improve male participation in the study, and this gain warrants further replication and investigation in future evaluations of social media recruitment strategies, particularly to better understand individuals’ motivations for participating in research studies. Such information could assist in developing strategies to optimize the recruitment and representativeness of samples; this might be particularly important for studies where higher levels of participation of a known *high risk* group is important. As the functionality of Facebook is advancing rapidly, emerging marketing features such as Facebook pixels or machine learning algorithms could be tested in future studies to advance the optimization and cost-effectiveness of recruitment.

## Appendix

Multimedia Appendix 1 Overview of advertisements across panels and advertisement performance metrics.

## Abbreviations

CTR: click through rate
NSW: New South Wales
