# Supplementary material for: Performance, Cost-Effectiveness, and Representativeness of Facebook Recruitment to Suicide Prevention Research: Online Survey Study
Source: JMIR Ment Health. 2020 Oct 22;7(10):e18762. doi: 10.2196/18762 (PMC7644381; doi:10.2196/18762)
Supplement: Multimedia Appendix 1 [file mental_v7i10e18762_app1.docx]

**Multimedia Appendix 1.** Overview of advertisements across panels and advertisement performance metrics.

| **Panel 1 recruitment: gender-neutral advertisements** | | |
| --- | --- | --- |
| Performance indicators | 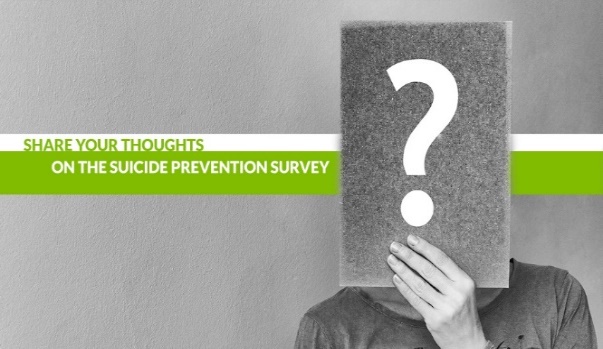  Advertisement 1: *LifeSpan is Australia's biggest ever suicide prevention trial, and we want to hear the thoughts of people living in the Murrumbidgee region to help make it better.  Take part now and help stop suicide in its tracks* | 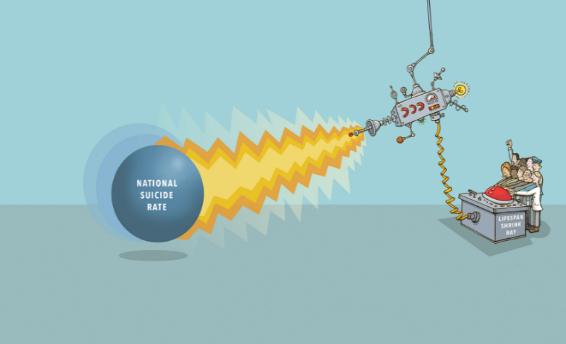  Advertisement 2: *The Murrumbidgee region is part of Australia's largest ever suicide prevention trial, and we need your help to make it better.*  *Take the survey and let us know what you know about suicide prevention* |
| Reach | 2596 | 865 |
| Cost per survey completion | $3.01 (US $2.16) | $4.79 (US $3.43) |
| **Panel 2 recruitment: gender-specific advertisements** | | |
|  | 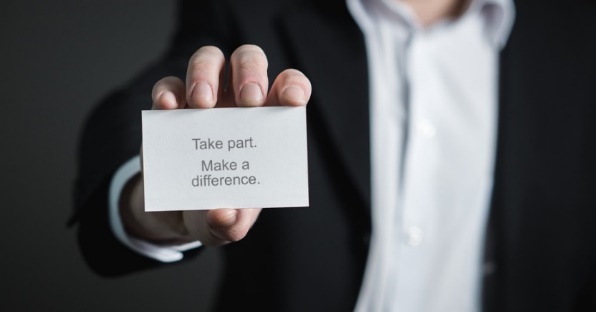  Advertisement 3: *The Murrumbidgee region is part of Australia's largest ever suicide prevention trial, and we need the voice of local blokes to help make it better.  Take the survey and let us know what you know about suicide prevention.* | 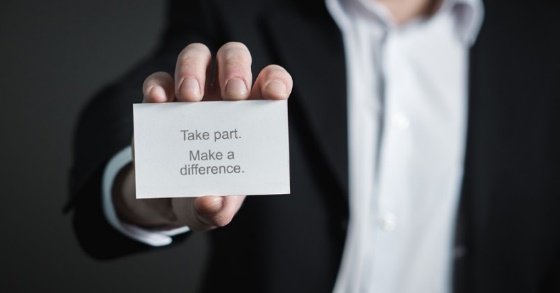  Advertisement 4: *We need men living in the Murrumbidgee region to help us with LifeSpan—Australia's largest ever suicide prevention trial.  Taking part in the survey today can help change life for countless people tomorrow.* |
| Reach | 6 678 | 2 141 |
| Cost per survey completion | $2.36 | $1.83 |
